# Provision and related factors of end-of-life care in elderly housing with care services in collaboration with home-visiting nurse agencies: a nationwide survey

**DOI:** 10.1186/s12904-021-00847-7

**Published:** 2021-09-30

**Authors:** Sakiko Fukui, Naoko Otsuki, Sumie Ikezaki, Hiroki Fukahori, Saori Irie

**Affiliations:** 1 Department of Home Care nursing, Graduate School of Health Care Sciences, Tokyo Medical and Dental University, 1-5-45 Yushima, Tokyo 113-8519 Bunkyo-ku, Japan; 2 Division of Health Sciences, Graduate School of Medicine, Osaka University, 1-7 Yamadaoka, Suita City, Osaka 565-0871 Japan; 3 Division of Health Promotion Nursing, Graduate School of Nursing, Chiba University, 1-8-1 Inohana, Chuo-ku, Chiba City, Chiba 260-8672 Japan; 4 Faculty of Nursing and Medical Care, Keio University, 4411 Endo, Fujisawa City, Kanagawa 252-0883 Japan

**Keywords:** Care facilities, Collaboration, End-of-life care, Home-visit nursing, Older people

## Abstract

**Background:**

Japan has the largest population of older adults in the world; it is only growing as life expectancy increases worldwide. As such, solutions to potential obstacles must be studied to maintain healthy, productive lives for older adults. In 2011, the Japanese government has started a policy to increase “Elderly Housing with Care Services (EHCS)”, which is one of a private rental housing, as a place where safe and secure end-of-life care can be provided. The government expect for them to provide end-of-life care by collaborating with the Home-Visit Nursing Agencies (HVNA). The purpose of this study is to clarify the situation of the end-of-life care provision in EHCS in collaboration with HVNA and to examine the factors that associate with the provision of the end-of-life care in EHCS.

**Methods:**

A two-stage nationwide survey (fax and mail surveys) were conducted. Of the 5,172 HVNA of the National Association for Visiting Nurse Services members, members from 359 agencies visited EHCS. Logistic regression analysis was conducted with the provision of end-of-life care to EHCS in 2017 as the dependent variable, and the following as independent variables: characteristics of HVNA and EHCS; characteristics of residents; collaborations between HVNA and EHCS; and the reasons for starting home-visit nursing.

**Results:**

Of the 342 HVNA who responded to the collaborations with EHCS, 21.6% provided end-of-life care. The following factors were significantly associated with the provision of end-of-life care to inmates in elderly care facilities: being affiliated with a HVNA, admitting many residents using long-term care insurance, collaborating with each other for more than three years, and started visiting-nurse services after being requested by a resident’s physician.

**Conclusions:**

This study clarified the situation of the provision of end-of-life care in EHCS in collaboration with HVNA and the related factors that help in providing end-of-life care in EHCS.

**Supplementary Information:**

The online version contains supplementary material available at 10.1186/s12904-021-00847-7.

## Background

Across the entire world, population aging is occurring more rapidly than it has in the past [1]. The number of people aged 60 years or older will increase from 900 million to 2 billion between 2015 and 2050 (12% to 22% of the total global population) [1].

Referencing a theoretical framework like “age-friendly communities (AFCs)” is important to construct a support system to respond to the growing population of older people around the world. AFCs typically focus on eight core community features to support the health for older people: housing, transportation, social participation, respect and social inclusion, civic participation and employment, communication and information, community support and health services, and outdoor spaces and buildings [2, 3]. It is crucial to pinpoint which of these eight features are needed to make AFCs depending on the development status of each country.

As the aging population increases, so does the number of deaths; specifically, the deaths of nursing home residents, and the deaths of older adults who reside at home in developed countries. We believe it is important to focus on the developed countries and on the method to construct an end-of-life care providing system for the older adults, focusing on “housing” among eight above-mentioned core features so that each older individual can spend their remaining years in good health whether they live in nursing homes or at home [4].

Japan has had the world’s highest population aging rate since 2004. In 2018, the per capita proportion of people aged 65 years and older reached the highest level at 27.7% [5]. Annual deaths in Japan have peaked to 1.3 million, with a mortality rate (per 1000 persons) of 10.8 [6]. Of these, 74.8% occurred in medical institutions, 13.2% in homes, and 10.0% in nursing homes, such as public long-term care facilities [7]. While the proportion of people dying in nursing homes has consistently increased over the past few years, the proportion of those in medical institutions began to decrease after reaching a peak of 82.4% in 2005 [6]. Once government reforms reduced the availability of hospital beds, the proportion of people dying in nursing homes is expected to increase even further [8]. Contrastingly, a recent report found that a total of 295,237 people are currently on a waiting list for a public nursing home, which is intended to act as an end-of-life home [8].

There have been growing concerns regarding the increasing number of older adults on these waiting lists. Moreover, the revision of the Long-Term Care Insurance Law in 2015 made it more difficult for the prospective residents to be admitted to a public nursing home, since the updated law only allows admittance to those who are certified as a long-term care need level three or above, with levels ranging from 1 (needed partial assistance) to 5 (bedridden). Under such circumstances, EHCS, which are privately run facilities, may receive older adults on such a waiting list.

The long-term care insurance (LTCI) system was introduced in 2000 in Japan to address the demands of older adults with disabilities based on the concept of a user-oriented social insurance system with support for independence. Older adults with a certification for LTCI service needs can utilize facility services, in-home services, and community-based services depending on their physical and cognitive impairments. In this system, every citizen aged 40 years or above pays premium, and the taxes are derived from the national government (25%), prefecture (12.5%), and municipality (12.5%). Older adults who are certified for the LTCI service pay a 10%-30% copayment for services; the remaining 70%-90% is covered by the LTCI budget [9].

In 2011, the Japanese government formulated its own EHCS system. It was originally meant to function as a housing facility with no assigned medical staff [10]. Here, older residents could receive lifestyle support services centered on supervision. However, if they needed direct care, they had to contact an external agency. Therefore, outsourcing healthcare from external agencies that employ numerous medical staff was required [11–13].

Subsequently, an increasing number of private enterprises have opened their own EHCS, which increased the number of EHCS from 70,999 in 2012 to 255,062 in 2020 [14]. Those aged 60 years or older can be admitted to these EHCS irrespective of their long-term care-need level. The EHCS are intended to be an end-of-life shelter for older people; and their increased availability may lead to wider end-of-life care options for them.

Contrarily, a 2012 survey found that only 8.8% of the EHCS provided end-of-life care; of these, 98.0% collaborated with external healthcare professionals. Additionally, a 2016 survey [15] found that, a high proportion of EHCS indicated that they did not provide end-of-life care because no nursing staff was available during the night shift (29.2%). In the same survey, 19.2% of the participants indicated that they did not provide end-of-life care due to nursing staff shortages. The above findings suggest that to increase the availability of end-of-life care in EHCS, it is essential for them to collaborate with external healthcare professionals, including home-visiting nurses.

Earlier studies of end-of-life care in EHCS, as well as the relationship between EHCS and home-visit nursing agencies (HVNA), indicate the following: the need for instruction by physicians and nurses to residential care staff [16]; the need to provide nursing-related methods and support to residential care staff [17]; and the need to collaborate with visiting nurses to provide palliative care [18]. Furthermore, another study found that elderly housings lacking staffed nurses did not have a clear approach to end-of-life care [19]. These findings focused on the need for multidisciplinary collaboration and enhancement of support systems. We believe that one of the important problems in elderly housing is a shortage of nurses [19] and it is crucial to find out the way to maintain a system for supporting end-of-life care by nurses from a medical professional perspective in elderly housing.

In this study, we focused on EHCS within elderly housing with good medical economic efficiency to efficiently distribute limited medical and human resources in an aging society. As EHCS is a relatively new service, information available on the actual state of end-of-life care is scarce. Thus, we conducted a nationwide survey of HVNA to gain a better understanding of end-of-life care provided by EHCS. Through this research, we believe that we can provide suggestions for enhancing the end-of-life care system not only in Japan, but also in other countries where the population of older adults is increasing.

Through this study, we aimed to clarify the situation of the end-of-life care provision in EHCS in collaboration with HVNA and examine the associations between the provision of end-of-life care in EHCS in collaboration with HVNA and the following factors: characteristics of HVNA, EHCS and residents; and reasons for starting home-visit nursing.

## Methods

### Study methods

This study was conducted in two stages: a fax survey (Stage 1) and a mail survey (Stage 2). From September to December 2017, a survey form was faxed to and collected from a total of 5,172 HVNA across Japan. The surveyed agencies were members of the National Visiting Nurse Association and accounted for more than 50% of the total 10,305 HVNA in 2017 [20]. Of these, 1,424 reported that they visited more than one EHCS. Thereafter, a mail survey of these 1,424 agencies was conducted from November 2017 to February 2018.

The reason for adopting the two processes is that HVNA, especially those that visit EHCS, have not been sufficiently popularized among long-term care insurance users, and there were no prior findings of the actual situation. As such, as a first step, we comprehended the number and percentage of HVNA visiting EHCS. Thereafter, as the second step, we designed a survey to reduce selection bias by distributing all questionnaires on the content of end-of-life care to HVNA visiting EHCS.

### Survey items

We measured the following characteristics of HVNA through questions gauging the number of staff (nurses, assistant nurses, rehabilitation workers), service users, visits (long-term care insurance, medical insurance), and residents where end-of-life care was provided at an EHCS. As for residents who received end-of-life care for which answers were requested in questionnaire, were defined as those who were diagnosed within six months of prognosis by the physician in charge. We listed the response by the participants about the resident who died most recently, in the category of “the characteristics of residents.”

This study is a policy research conducted by researchers in 2018 on behalf of the Japanese Ministry of Health, Labor and Welfare. In selecting the survey items, we referred of the framework of the concept of Avedis Donabedian [21], who proposed a medical quality evaluation model, InterRAI [22, 23], OASIS [24], and the Japanese Nursing Association's "Improvement of labor and nursing quality (DiNQL)." [25]

The characteristics of EHCS with home-visiting nurses were explored through questions on the following topics: being affiliated with HVNA; the number of residents receiving visiting nurse services for the previous month; the cumulative total visits to the EHCS for the previous month; the period from the first nurse’s visit to the EHCS to the time when the survey was conducted; the reason why visiting nurse services were introduced to the EHCS; and the collaboration status between EHCS staff and nurses. For the question on the reason for introducing visiting nurse services to an EHCS, the participants were given five choices for answers (requested by: EHCS staff; physician; hospital staff; care manager; and original HVNA user who admitted at EHCS).

### Analysis methods

For this study, basic information items related to EHCS, as mentioned above, that received visiting nurse services was set as independent variables and the provision of end-of-life care at EHCS for the previous year as the dependent variable. A univariate logistic regression to confirm the one-to-one associations with each independent and the dependent variable was performed. A Pearson correlation coefficient analysis was then performed between all independent variables, and those that were highly correlated (>0.7) were excluded to avoid multicollinearity. Subsequently, we performed a multivariate logistic analysis by throwing all the nine independent variables used in a univariate analysis. Residents using medical insurance were excluded from the analysis as the number of residents using medical insurance were insufficient for an analysis to be performed.

The sample size of this study was set with 10 times the number of independent variables by referring to the papers by Peduzzi et al. [26, 27]

Variables were selected after confirming the internal correlation between each. SAS ver. 9.4 was used for analysis with a 5% significance level (two-tailed).

## Results

### Characteristics of HVNA

**Fig. 1:**
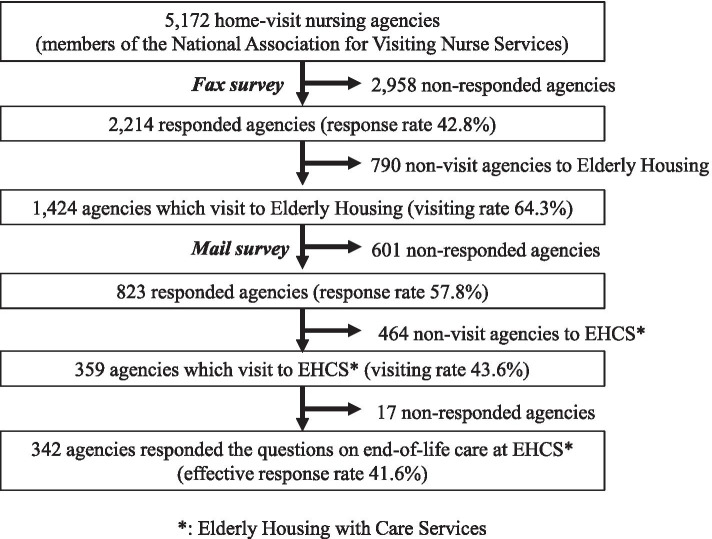
Flow chart of participants

Responses were obtained from 2,214 of the 5,172 agencies in the fax survey (response rate: 42.8%). Among these, 1,424 visited aged care facilities, including all public and private care facilities (visiting rate 64.3%). Among these, 823 agencies responded to the mail survey (response rate: 57.8%). Out of the 823, 359 agencies (43.6%) had nurses visiting the EHCS. Data from 342 facilities (i.e., the number of cases that end-of-life care was provided at EHCS for the previous year) were analyzed (effective response rate: 41.6%) (Fig. 1). Responses from the 342 HVNA described the characteristics of one of the EHCS that they visited, as well as those of EHCS residents who used services provided by the visiting nurses (Table 1).

**Table 1:** Characteristics of home-visit nursing agencies (HVNA) and residents of ‘Elderly Housing with Care Services’ (EHCS) (n=342)

Variables	Mean±SD^a^ or n(%)	
*Characteristics of HVNA*		
The number of staffs (full-time/part-time)	Nurses	4.4±2.6 / 2.9±3.3
	Associate nurses	0.1±0.5 / 0.2±0.7
	Rehabilitation workers^b^	1.7±3.0 / 1.3±2.4
The number of users /visits for last month	Users using long-term care insurance	63.5±55.5 / 379.2±362.1
	Users using medical insurance	30.7±38.8 / 222.1±215.0
End-of-life care in a collaborated EHCS for last year (presence)	Number of residents who died	0.7±3.1
	0	268 (78.4)
	1	36 (10.5)
	2	13 (3.8)
	3	12 (3.5)
	4	4 (1.2)
	>=5	9 (2.6)
	Maximum	48
Staff’s occupation in a collaborated EHCS (multiple answers)	Nurse (full-time)	106(31.0%)
	Nurse (part-time)	81(23.7%)
	Long-term care workers	225(52.1%)
	Rehabilitation workers^b^	34(9.9%)
	Care Managers	150(43.9%)
	Administrator	166(38.4%)
	Facility manager	133(38.9%)
*Characteristics of residents*^c^*of EHCS*		
Age of residents in a collaborated EHCS	50’s	5(1.5%)
	60’s	29(8.5%)
	70’s	58(17.0%)
	80’s	143(41.8%)
	90’s	81(23.7%)
	No answer	26(7.6%)
Sex of residents in a collaborated EHCS	Male	100(29.2%)
	Female	219(64.0%)
	No answer	23(6.7%)
Care-need level of residents in a collaborated EHCS	Care-support level 1	9(2.6%)
	Care-support level 2	13(3.8%)
	Care-need level 1	50(14.6%)
	Care-need level 2	52(15.2%)
	Care-need level 3	54(15.8%)
	Care-need level 4	55(16.1%)
	Care-need level 5	68(19.9%)
	In the application	2(0.6%)
	No answer	39(11.4%)

^a^SD: standard deviation

^b^physical therapist, occupational therapist, speech therapist

^c^: about the most recent resident who received end-of life care last year in responded EHCS

For the question on the provision of end-of-life care at EHCS for the previous year, 74 facilities responded “yes” (21.6%), whereas 268 responded “no” (78.4%). The mean number of residents who died was 0.7±3.1. For the question on collaboration with EHCS staff, 225 agencies (52.1%) indicated that the visiting nurses collaborated with long-term care workers, and 150 (43.9%) indicated that the visiting nurses collaborated with the care managers (Table 1).

We also showed the comparison of the provision of end-of-life care at EHCS by the characteristics of HVNA and EHCS (Table 2).

**Table 2:** Comparison of the provision of end-of-life care at EHCS^a^ by the characteristics of HVNA^b^ and EHCS

Variables		Total n=342 n (%) or Mean±SD	Provided EOL^c^ care n=74 n (%) or Mean±SD	Not provided EOL care n=268 n (%) or Mean±SD
Affiliation with a EHCS	Affiliated	52(15.2%)	26(35.1%)	26(9.7%)
	Not affiliated	278(81.3%)	45(60.8%)	233(86.9%)
	Unknown	12(3.5%)	3(4.1%)	9(3.4%)
The number of residents /visits for last month in a collaborated EHCS	Residents using long-term care insurance	5.2±9.5	10.9±13.4	3.6±8.0
	Residents using medical insurance	0.03±0.27	0±0	0.03±0.30
	Residents paying out-of-pocket	26.6±74.9	62.5±112.8	17.2±61.5
	Visits using long-term care insurance	32.7±65.3	65.1±98.8	23.2±51.6
	Visits using medical insurance	2.4±7.9	3.4±5.7	2.1±8.4
	Visits paying out-of-pocket	0.2±2.0	0±0	0.3±2.3
Duration of collaboration with the EHCS	Less than 3 years	231(67.5%)	36(48.6%)	195(72.8%)
	More than 3 years	111(32.5%)	38(51.4%)	73(27.2%)
The reason for starting home-visit nursing for a EHCS resident (multiple answers)	Requested by a EHCS staff	95(27.8%)	35(47.3%)	60(22.4%)
	Requested by a physician	126(36.8%)	39(52.7%)	87(32.5%)
	Requested by a hospital staff	50(14.6%)	13(17.6%)	37(13.8%)
	Requested by a care manager	212(62.0%)	45(60.8%)	167(62.3%)
	HVNA user and admitted to a EHCS	75(21.9%)	10(13.5%)	65(24.3%)
	Unknown	1(0.3%)	0(0%)	1(0.4%)
	Others	22(6.4%)	3(4.1%)	19(7.1%)

^a^ elderly housing with care services

^b^ home-visit nursing agencies

^c^ end-of-life

### Associations between the provision of end-of-life care at EHCS and the characteristics of EHCS

#### Univariate analysis

Significant differences were observed in the items that were associated with the provision of end-of-life care at EHCS for the previous year: being affiliated with an HVNA, admitting many residents using long-term care insurance, admitting that many residents paying out-of-pocket, collaborating with each other for more than three years, visiting nurse services being requested by an EHCS staff, visiting nurse services being requested by a physician, and visiting nurse services being introduced as an existing service user was admitted to the EHCS (Table 3).

**Table 3:** Associations between the provision of end-of-life care at EHCS^a^ and its characteristics: logistic regression analyses

Independent variables		Univariate		Multivariate ^c^	
		Odds Ratio(95%CI)	p-value	Odds Ratio(95%CI)	p-value
Affiliation with an EHCS^a^ (affiliated)		5.18(2.76-9.78)	<.001	3.13(1.30-7.54)	0.01
The number of residents for last month in a collaborated EHCS	Residents using long-term care insurance	1.07 (1.04-1.11)	<.001	1.08(1.04-1.13)	<.001
	Residents paying out-of-pocket	1.003(1.001-1.005)	0.03	1.001(0.99-1.01)	0.63
Duration of collaboration with a EHCS: more than 3 years		2.88 (1.69-4.92)	<0.01	2.39(1.11-5.15)	0.02
The reason for starting home-visit nursing for a EHCS resident	Requested by a EHCS staff	3.15 (1.83-5.45)	<0.01	1.31(0.59-2.84)	0.49
	Requested by a physician	2.35 (1.39-4.01)	<0.01	2.75(1.29-6.00)	0.01
	Requested by a hospital staff	1.33 (0.64-2.60)	0.42	1.48(0.56-4.29)	0.44
	Requested by a care manager	0.93 (0.54-1.61)	0.79	1.14(0.50-2.54)	0.74
	HVNA^b^ user and admitted to a EHCS	0.48 (0.22-0.96)	0.05	2.65(0.99-8.18)	0.07

^a^ elderly housing with care services

^b^ home-visit nursing agencies

^c^ In a multivariate analysis the same nine variables with a univariate analysis were thrown as independent variables.

### Logistic regression analysis

The following characteristics were confirmed to be associated with the provision of end-of-life care to at least one case at EHCS that a nurse visited in the previous year: being affiliated with an HVNA, admitting a large number of residents using long-term care insurance, collaborating with each other for more than three years, and starting to use home-visit nursing services after a referral from a resident’s physician. Additionally, the variance inflation factor between the related factors was within the range of 1.021–1.300. No multicollinearity was observed (Table 3).

## Discussion

This study clarified the situation of the end-of-life care provision in EHCS in collaboration with HVNA and the related factors that helps to promote the provision of end-of-life care in EHCS. The findings clarified the following four associated factors: being affiliated with a HVNA, admitting a large number of residents using long-term care insurance, collaboration between the HVNA and EHCS for at least three years, and introducing visiting nurse services by referral from the resident’s physician.

This study also clarified that a relatively low proportion of participating HVNA (21.6%) provided end-of-life care in EHCS. A national survey showed that 20.4% of all EHCS in Japan have provided end-of-life care [15]. Increasing the proportion of EHCS that provides end-of-life care may be one of the solutions for HVNA who have not yet collaborated with EHCS to start collaborating with EHCS to support end-of-life care residents, by strengthening the related factors discussed below.

In regard to one of the related factors to promote the end-of-life care provision in EHCS, being affiliated with HVNA, a previous study found the importance of affiliations between EHCS and HVNA to provide end-of-life care, and the fact that the EHCS is not necessarily required to employ nurses [18]. There might arise a situation when a nurse is required to be available on-site 24 hours a day at the EHCS. Considering how difficult it may be to increase the number of EHCS that employ nurses providing effective end-of-life care, it would be more realistic for policymakers and facility managers to develop a system that strengthens the collaboration between the EHCS and HVNA. A good solution may be not only to enhance these collaborations but also to encourage ones between EHCS and those not currently affiliated with HVNA. The results also suggest that end-of-life care is likely provided at EHCS with a large number of residents using long-term care insurance. This could be because these facilities have a large number of residents, and thus offer many opportunities for end-of-life care. Additionally, 11% of EHCS were designated as specialized facilities [14], where there were many long-term care insurance users. This is because such facilities are residential facilities where personnel like nursing staff, equipment standards authorized by the local government in the long-term care insurance (LTCI) system, and the service fee for direct care are included in the contract fee [28]. A previous study indicated that the need for visiting nurse services is related to the degree of dependency of the residents on healthcare [29]. Furthermore, our results suggest that such a necessity is also related to long-term care requirements. In fact, while the mean long-term care level for general EHCS residents was 1.76 (range: care level 1 [needed partial assistance] to level 5 [bedridden]) [30], the mean care level for residents to whom the nurses visited was 3.5, suggesting that the residents with a high long-term care need level are likely to use home visiting nurse services. Thus, visiting nurse services are pivotal to efficient end-of-life care for residents of EHCS with high medical and long-term care requirement levels. However, further research on the characteristics of EHCS is required to gain a clearer perspective on it.

Of the EHCS included in this study, those who had collaborated with HVNA for at least three years were found to be more likely to provide end-of-life care. A relationship of trust is essential for collaboration between long-term care workers and nurses [31, 32]. Visiting nurses who have built a favorable relationship with long-term care workers tend to proactively collaborate with them [33]. Thus, a relationship of trust is also important when the EHCS and HVNA collaborate to provide end-of-life care. Our results suggest that a substantial amount of time is required to build a foundation that underpins such a relationship.

Moreover, we found that end-of-life care is likely to be provided at EHCS where visiting nurse services were introduced after being requested by a resident’s physician. In 2005, the Japanese government announced a plan to improve the cost-effectiveness of health services as part of the Outline of Medical Care System Reform [34]. Reducing the mean number of days of hospitalization was one of the measures implemented in the plan [34]. As a previous study indicated, our study also found that the greater the patient’s healthcare dependency level, the greater the need for visiting nurse services [29]. Our results suggest that visiting nurse services are increasingly necessary in end-of-life care with the rapid population aging. Unfortunately, a survey found that even physicians and related health care professionals, as well as community residents, were unaware of visiting nurse services [35]. It presented several cases where the condition of a resident worsened due to delays in visiting nurse services that can be attributable to a lack of awareness. Specifically, to increase the availability of end-of-life care, it is necessary to inform physicians that visiting nurse services have the capacity to address health-related needs in end-of-life care. This may help physicians deepen their understanding of the necessity of visiting nurse services and, more importantly, gain a new perspective. It may be necessary to focus not only on EHCS staff, but also on physicians who are proactive, while introducing visiting nurse services to EHCS. Furthermore, it may be advantageous to encourage residents to choose such proactive physicians as their primary physicians.

## Limitations

This study has several limitations. First, a small number of HVNA are currently visiting EHCS, leaving a gap in the end-of-life care experience between the facilities. Thus, the results of this study cannot yet be generalized. As the result’s applicability to other settings is not possible, the future investigation is required. Second, respondents of this survey were nurses of HVNA, so we obtained responses about the characteristic of EHCS by either nurses’ referring to the contract record with EHCS or requesting inquiries to the partner EHCS. Therefore, some characteristics of EHCS in this survey items were inadequate. Third, this study focused on the provision of end-of-life care only from the perspective of HVNA. Thus, further research is required to examine the importance of visiting nurse services from the perspective of EHCS. Further studies should be conducted to explore the following areas: differences in the experience of end-of-life care between EHCS, details of collaboration, and factors related to end-of-life care. Fourth, we could not consider many confounders such as the degree of dependency on healthcare and diseases that the residents had in the analysis of this study. Further research considering these confounders is necessary. Finally, since this study used a cross-sectional study design, it is not possible to state a causal relationship. Future studies should accumulate research findings while also ensuring that increasing frequency with which EHCS use visiting nurse services.

## Conclusions

This study clarified the situation of the end-of-life care provision in EHCS in collaboration with HVNA and the related factors to provide the end-of-life care in EHCS: being affiliated with an HVNA; admitting a large number of residents using long-term care insurance; collaboration between the HVNA and EHCS for at least three years; and introducing visiting nurse services by referral from the resident’s physician. To strengthen the providing system of the end-of-life care at EHCS, these factors might be important: keeping opportunities for collaborations between EHCS staff and visiting nurses; continuing to live in EHCS receiving the long-term care service; maintaining collaborations between EHCS staff and HVNA nurses for at least three years; and promoting understanding of HVNA by a resident ’s physician. We hope that our study adds to the research that will lead to the creation of an effective, sustainable model of care that will allow the elderly to live out the rest of their lives with dignity and good health.

## Supplementary Information

**Additional file 1.**

## Data Availability

The datasets used and analyzed during the current study are available for further use (See Additional file 1). Corresponding author SF should be contacted if someone wants to request the data. Although public access to the database is closed, everyone can access the database of this study from the corresponding author on reasonable request without an administrative permissions to access the raw data. All data generated or analyzed during this study are included in this published article.

## Abbreviations

EHCS: Elderly Housing with Care Services
HVNA: Home-Visit Nursing Agencies
